# Depletion of the oncoprotein Bcl-3 induces centrosome amplification and aneuploidy in cancer cells

**DOI:** 10.1186/1476-4598-9-223

**Published:** 2010-08-24

**Authors:** Ruben Zamora, Magali Espinosa, Gisela Ceballos-Cancino, Blanca Segura, Vilma Maldonado, Jorge Melendez-Zajgla

**Affiliations:** 1 Biotechnology Unit, Grupo Farmacéutico Neolpharma, (Boulevard de los Ferrocarriles 277), Mexico City (02300), Mexico; 2 Cancer Functional Genomics Laboratory. National Institute of Genomic Medicine. (Periferico Sur 4124), Mexico City (01900), Mexico; 3 Gene Therapy Laboratory, Laboratorios Alpharma, (Renato Leduc 363) Mexico City (14050), Mexico; 4 Molecular Biology Laboratory. Instituto Nacional de Cancerologia, (Av. San Fernando 22) Mexico City (14080), Mexico

## Abstract

Bcl-3 is an atypical member of the inhibitor of NF-kappa B family of proteins since it can function as a coactivator of transcription. Although this oncogene was described in leukemia, it is overexpressed in a number of solid tumors as well. The oncogenic potential of Bcl-3 has been associated with its capacity to increase proliferation by means of activating the cyclin D1 promoter and to its antiapoptotic role mediated by the inhibiton of p53 activity. In the course of dissecting these properties, we found that depleting Bcl-3 protein using shRNAs induce a decrease of proliferation and clonogenic survival associated with the induction of multinucleation and increased ploidy. These effects were associated with a DNA damage response, a delay in G2/M checkpoint and the induction of centrosome amplification

## Findings

The Bcl-3 oncogene was first identified in a subset of patients with chronic lymphocytic leukemia (CLL) in the region adjacent to the t(14;19)(q32;q13) translocation [1,2]. Bcl-3 is a member of the NF-kappa B inhibitor family, which includes I-kappa B alpha, beta, epsilon and zeta. NF-kappa B is a pleiotropic transcription factor involved in several physiological and pathological processes such as inflammation, immune response and cancer. NF-kappa B inhibitory proteins act to suppress NF-kappa B activity by blocking the nuclear translocation of this factor, thus preventing the transcription of target genes. The protein encoded by Bcl-3 is atypical among inhibitory proteins because it is able to activate transcription [3-5]. This effect is mediated by its association with the NF-kappa B homodimers, p50 and p52, along with coactivators such as CBP/p300, SRC-1 and Tip60 [4,6]. While Bcl-3 overexpression was originally found to be associated with CLL and lymphomas, recent reports have shown that this oncogene is overexpressed in several solid tumors, such as breast [7], nasopharyngeal [8] and endometrial [9] carcinomas.

Although some advances have been made in defining the cellular and molecular effects of Bcl-3, its precise role in carcinogenesis is still unclear. Earlier work by Westerheide et al. showed that in breast epithelial cells, Bcl-3 is able to act as a coactivator with NF-kappa B p52 homodimers to directly activate the cyclin D1 promoter, so direct effects on the cell cycle could be responsible for its oncogenic effects [10]. Also supporting this line of evidence is the finding that the tumor suppressor CYLD blocks cyclin D1 expression by inhibiting Bcl-3 signaling [11]. An additional role for Bcl-3 in carcinogenesis has recently been suggested by Kashatus et al., who demonstrated that after DNA damage, Bcl-3 is required for the induction of Hdm2 gene expression and the suppression of persistent p53 activity [12]. These results suggest that the oncogenic role of Bcl-3 could be mediated by a p53-dependent pathway, possibly by preventing the apoptosis of cells with damaged DNA.

**Figure 1:**
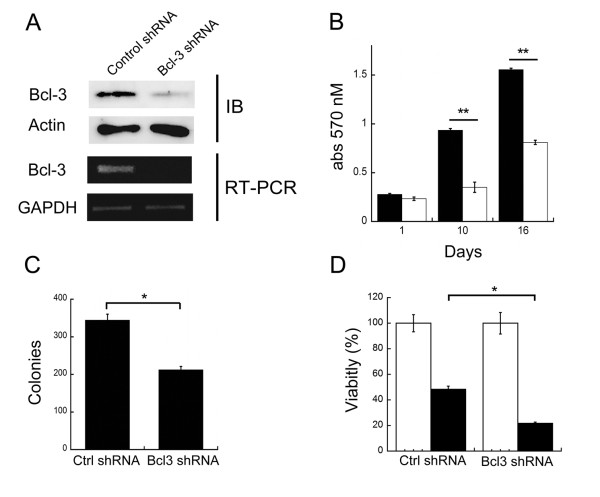
**Knockdown of Bcl-3 in HeLa cervical cancer cells**. HeLa cells were infected with retroviral particles packaged with constructs derived from pSIREN vector (Clontech). These vectors codifies for a double-stranded short hairpin RNA (shRNA) directed toward Bcl-3 under the human U6 promoter. Cells were selected with puromycin for 1 week and protein (upper panel A) or RNA (lower panel A) analyzed. B) HeLa cells were grown in DMEM plus 8% fetal bovine serum for the times shown and viability assessed with the crystal violet method. Mean and standard deviation of the optical absorbance are shown. Black bars: cells expressing a control (scrambled) shRNA, white bars: cells expressing a Bcl-3 shRNA. C) Clonogenic assays. 2.5 × 10^3 ^cells were seeded in 100 mm petri dishes, cultivated for 16 days, fixed and stained with crystal violet. Colonies were manually counted. D) HeLa cells were grown in DMEM plus 8% fetal bovine serum, exposed to ultraviolet B light (280 nm) for 45 seconds (≈ 300 J/m^2 ^) and viability assessed by crystal violet staining 24 hours later. Mean and standard deviation of the optical absorbance are shown. Black bars: cells expressing a control (scrambled) shRNA, white bars: cells expressing a Bcl-3 shRNA. All the assays were performed by triplicate in three separated experiments. Ctrl: control. IB: Immunoblot. *p < 0.05, **p < 0.01 using student's T test.

In order to dissect the contribution of apoptosis and proliferation to the oncogenic effects of Bcl-3, we produced stable cell lines expressing small-interfering RNAs (siRNAs) directed toward the oncoprotein (Fig. 1a and Additional file 1, Fig. S1). As expected, based on the previously mentioned reports, downregulation of Bcl-3 levels had a significant negative impact on the growth of these cells. Fig 1b shows a significative reduction in the number of Bcl-3 knockdown cells in a time course experiment (Fig. 1b). In addition, a clonogenic assay, which assesses the reproductive success of the cells, showed a 40% decrease in the number of colonies formed (Fig. 1c). Since the decrease in cellular growth and reproductive efficiency could also be due to cell death, we measured the rate of spontaneous apoptosis via annexin V (not shown) and TUNEL assays. Interestingly, we did not observe differences in apoptotic rates between cells with down regulated Bcl-3 and the control cells (Additional file 2, Fig. S2). This is an unexpected finding, since it has been shown that Bcl-3 is able to suppress p53 activation and apoptosis after DNA damage [12]. This discrepancy could be due to the fact that HeLa cervical cell line express the papillomavirus E6 oncoprotein, which destabilizes p53 and, therefore, should be resistant to the apoptotic effects of Bcl-3 [13].

This result excluded cell death as the mechanism for the observed effect of Bcl-3 on the growth and clonogenic survival of HeLa cells. Nevertheless, further analyses showed that depletion of Bcl-3 rendered cells more sensitive to stress-induced apoptosis (Fig. 1D and Additional file 2, Fig. S2 A and C).

**Figure 2:**
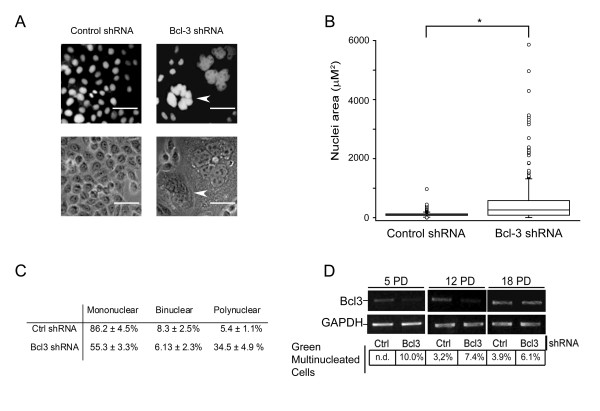
**Bcl-3 knockdown cells are multinucleated**. A) Nuclei of HeLa cells expressing a control shRNA (left panels) or Bcl-3 shRNA (right panels) as described in Fig. 1 were stained with ethidium bromide after five population doublings. Arrow shows a multinuclear cell. B) Box-and-whisker plots of nuclear area from a thousand nuclei of HeLa cells expressing a control shRNA or Bcl-3 shRNA as described in Fig. 1. Nuclei were stained with ethidium bromide. These results were obtained in the described cells after five population doublings. The plots show quartiles, sample minimum and maximum. *p < 0.01 by Wilcoxon rank test. C) Table showing the percentage and standard deviation of mono, bi or multinuclear cells derived from two hundred cells counted in three different preparations of the described cells after five population doublings. D) HeLa cells were transiently transfected with a plasmid encoding a shRNA directed toward Bcl-3 and, along, the ZsGreen protein marker (plasmid pSIREN-RetroQ-ZsGreen, Clontech; CA, USA) (Bcl3). As control, a plasmid with encodes a scrambled shRNA and the same marker, was transfected (Ctrl). After 5, 12 or 18 population doublings (PD), green multinuclear cells were counted (percentage at the bottom) n.d.: not detected. RNA was also isolated from these passages and assayed for Bcl-3 expression by RT-PCR. Note that at the 18^th ^PD, the Bcl-3 knockdown effect was lost. Transfection efficiency was approximately 30%. The differences observed between the experiments in Fig. 1 and 2 were probably due to the lower efficiency of the knockdown strategy used (infection versus transfection).

**Figure 3:**
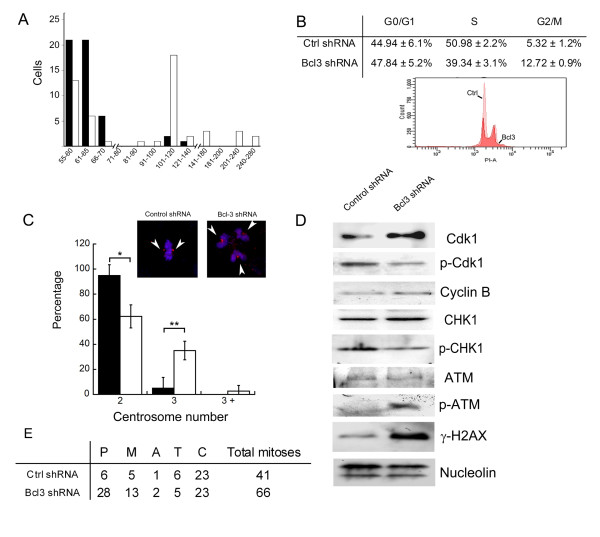
**Increased ploidy and centrosome amplification in Bcl-3-depleted cells**. A) Histogram showing HeLa cells stably expressing control (black bars) or Bcl-3 (white bars) shRNAs were treated with colcemide and subjected to a karyotype analysis. Careful examination of fifty karyograms was perfomed and chromosome counts obtained. B) Table showing percentage and standard deviation of cell populations in G1, S and G2/M examined by flow cytometry analysis. C) HeLa cells stably expressing control or Bcl-3 shRNAs stained with an anti- gamma tubulin antibody and visualized using fluorescence microscopy. The graph shows percentage of control (black bars) or Bcl-3-depleted (white bars) cells with 2, 3 or more than three centrosomes at mitosis. Two hundred and fifty fields were scored in three independent experiments. * p = 0.02; **p = 0.04 by Student's T test. Inset: representative image. Arrows show centrosomes. D) Western blot analyses of cell with downregulated Bcl-3 levels. E) Effect of the expression of a Bcl3 shRNA in the number of HeLa cells at the indicated cell cycle phase (number of cells/4000 cells). The cells were transfected with a plasmid that direct the expression of a Bcl3 or a control shRNA and, simultaneously, an Enhanced Green Fluorescent Protein as a marker. After 48 hours, the cells were stained with an antibody directed toward γ-tubulin, a Cy5-marked secondary antibody and DAPI. Phases were scored only from green cells and results were pooled from three separate experiments. P: Prometaphase, M: Metaphase, A: Anaphase, T: Telophase, C: Cytokinesis.

In order to investigate the possible mechanism of the decreased growth, we followed Bcl-3 knockdown cells and found, surprisingly, that more population doubling resulted in an increasing number of multinucleated cells, as assessed by fluorescent nuclear staining (Fig. 2a). The increase in multinuclear cells reached statistical significance after five passages (Fig. 2c). To assess if the changes were progressive and to provide additional support using a different expression approach, HeLa cells were transfected with plasmids encoding sequences for Bcl-3 and control shRNAs and followed for several passages. We found that Bcl-3-depleted cells decreased progressively from the culture (Fig. 2d), perhaps by dilution by non-transfected cells, thus precluding a more detailed analysis of the phenotype. A more careful examination using image analysis of fixed cells showed that Bcl-3-depleted cells were not only polynuclear, but also demonstrated a larger nuclear area (Fig. 2b). The larger area is suggestive of an increased cellular ploidy in Bcl-3-depleted cells. To investigate this possibility, we performed karyotype analyses. Fig 3 shows that HeLa cells are aneuploid by nature, with a chromosome number range between 55 and 140 and a modal number between 51 and 60 (Fig. 3a). In sharp contrast, we found that the downregulation of Bcl-3 induced an increase of the chromosome number range to 55-280, with a modal number between 101 and 120 (Fig. 3a). These results clearly showed that Bcl-3 is a key regulator of mitosis in HeLa cells.

Cell cycle checkpoints are essential for maintaining genetic stability. In response to DNA damage, cells arrest in G1/S or G2/M checkpoints for repair. Extended arrest in the G2/M checkpoint after DNA damage can induce centrosome amplification and failed cytokinesis by ATM-dependent and -independent signaling [13]. This phenomenon is thought to represent a mechanism for ensuring the reproductive death of cells that evade the spindle assembly checkpoint. To assess if Bcl-3-depleted cells were, indeed, arrested at the G2/M checkpoint, we performed flow cytometric analyses and found a significant increase in this population of cells relative to the control (Fig. 3b). In addition, we found a progressive increase in the mitotic index (4.27% in control cells versus 7.42% in Bcl3-depleted cells at 12 population doublings; 3.00% in control cells versus 10.41% in cells deficient for Bcl-3 at 18 population doublings). To provide further insight, we then analyzed cells transiently-transfected with a plasmid that expresses a Bcl3 shRNA and a green fluorescent marker (EGFP). Fig 3E shows that Bcl-3 depletion increased the number of cells in pro-metaphase and metaphase, suggesting that the multinucleation effect may be mediated by a faliure to satisfy the spindle assembly checkpoint. In support of this result, we found that cyclin-dependent kinase 1 (Cdk1) was hypophosphorylated and cyclin B expression increased in Bcl-3-depleted cells, a result consistent with the mitosis-promoting effect of this complex. In order to assess if G2/M arrest did, indeed, induce centrosome amplification, we stained the mitotic spindles using a tubulin antibody. As shown in Fig 3c, cells with downregulated Bcl-3 demonstrated a significant increase in centrosome numbers, thus, corroborating the amplification of centrosomes at mitosis. As expected, no differences were found at interphase in centrosome numbers (70% with 2 centrosomes and 30% with 1 centrosome in both Bcl-3 defficient and control cells). Finally, we assessed whether Bcl-3 depletion could induce a DNA damage response. As shown in Fig 3d, Western blot analysis demonstrated an increase in the phosphorylation of histone 2AX, a marker of DNA damage [14], as well as increased phosphorylation of the kinase responsible for this modification, ATM, in Bcl-3-depleted cells. Interestingly, phosphorylation of Chk1, an essential serine/threonine kinase required for the cell cycle checkpoint arrest after DNA damage [15], was reduced by Bcl-3 depletion. These results showed that Bcl-3 downregulation induces a DNA damage response that is uncoupled from the checkpoint-mediated cell cycle arrest.

Cancer cells frequently show amplification of centrosome number and aneuploidy. It has been shown that both processes could be linked to tumor progression, tumor regrowth and drug resistance [16]. Centrosome numeric aberrations arise as a consequence of failures in cytokinesis, which can be induced by abnormalities in the expression or regulation of several protein kinases and multiple genetic aberrations, in particular the loss of the tumor suppressor, p53 [17]. In this regard, it is thought that cumulative DNA damage or mutations in DNA repair genes are responsible for this phenomenon [18]. The presence of cancer cells with more than two centrosomes in clinical samples suggests the existence of mechanisms that allow cells to survive despite this amplification. In the present paper, we provide evidence that the depletion of Bcl-3 expression is associated with centrosome amplification and an endopolyploid phenotype. This phenotype could be due to a direct effect of this oncogene on centrosome amplification and cytokinesis aberrations or could be indirectly caused by an undescribed cell death function. In the latter case, the effects of Bcl-3 depletion should be due to the accumulation of aberrant cells in the culture. This is unlikely since we did not find evidence of increased cell death, and the growth and clonogenic potential of Bcl-3-depleted cells were also decreased. Alternatively, Bcl-3 could participate in the genesis of centrosome amplification and cytokinesis aberrations by disrupting the DNA damage response. This theory is supported by the findings of Boulton et al., who showed that the C. elegans Bcl-3 ortholog is involved in the DNA damage response [19]. In addition, Watanabe et al. showed that Bcl-3 binds to B3BP, a polynucleotide kinase with a MutS-related domain, which is thought to be involved in DNA repair [20]. Since Bcl-3 knockout mice do not show any gross defect in DNA damage response, the participation of this oncogene should be specific to cancer cells and probably requires *de novo *overexpression.

Recently, it has been postulated that the oncogenic potential of Bcl-3 could be related to the capacity of this proto-oncogene to suppress p53 activation. This finding implies that Bcl-3 action should be restricted to tumors harboring no p53 alterations. Although not exhaustively examined, our analyses of cultured cell lines do not show a correlation between p53 mutation status and Bcl-3 expression (Additional file 3, Fig. S3). In addition, the cell lines used in this study were derived from HeLa cells, which express papillomavirus protein E6 and, thus, have a disrupted p53 signaling pathway. Thus, Bcl-3 oncogenic potential could not only be limited to its effects on p53 activation, but to its effects on limiting cell cycle and chromosomal number abnormalities in p53-mutant cells. Additional experiments in mouse models are needed to explore this hypothesis.

In conclusion, the present paper shows that the depletion of Bcl-3 in cancer cells induces centrosome amplification and increases ploidy. Bcl-3 may be needed to overcome the deleterious effect of DNA damage on cancer cells with aberrant mitosis, and the importance of Bcl-3 could be exploited for the development of selective inhibitors of tumorigenesis.

## List of abbreviations

ATM: Ataxia Telangiectasia Mutated; Bcl-3: B-cell CLL/lymphoma 3; Chk1: Checkpoint homolog-1; CYLD: Cylindromatosis.

## Competing interests

The authors declare that they have no competing interests.

## Authors' contributions

RZ produced transgenic cell lines and most experimental data, ME produced transgenic cell lines and provided molecular data, GC produced cell death analysis and molecular data, BS provided western blot analyses, VM conceived the study and participated in its design, JM-Z conceived and coordinated the study and wrote the manuscript. All authors read and approved the final manuscript

## Supplementary Material

Additional file 1 **Figure S1. Morphology of MCF-7 Breast Cancer cells expressing a Bcl-3 shRNA**. Breast cancer cells present a similar morphology to HeLa cells after Bcl-3 depletion. Click here for file

Additional file 2 **Figure S2. Effects of Bcl-3 depletion on apoptosis**. Cells with Bcl-3 knockdown are more susceptible to apoptosis induced by ultraviolet light, as assessed by nuclear morphology, viability assays and TUNEL. Click here for file

Additional file 3 **Figure S3. Analysis of Bcl-3 mRNA expression of breast cancer cell lines by RT-PCR**. Bcl-3 steady-state levels in breast cancer cell lines with different p53 status (wild type or mutated) and expression. Click here for file
