# Supplementary material for: Depletion of the oncoprotein Bcl-3 induces centrosome amplification and aneuploidy in cancer cells
Source: Mol Cancer. 2010 Aug 24;9:223. doi: 10.1186/1476-4598-9-223 (PMC2933622; doi:10.1186/1476-4598-9-223)
Supplement: Additional file 1 — Figure S1. Morphology of MCF-7 Breast Cancer cells expressing a Bcl-3 shRNA. Breast cancer cells present a similar morphology to HeLa cells after Bcl-3 depletion. [file 1476-4598-9-223-S1.PDF]

Additional file 1. Figure S1.

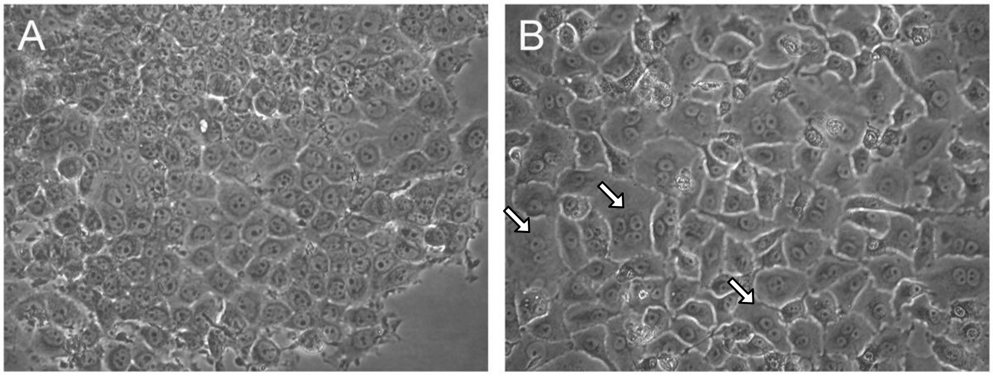

Morphology of MCF-7 Breast Cancer cells expressing a Bcl-3 shRNA. A) Cells transfected with a control shRNA. B) Cells expressing a shRNA directed against Bcl-3. Second passage after transfection. Note the increased nuclei number (arrows).
