# Supplementary material for: Depletion of the oncoprotein Bcl-3 induces centrosome amplification and aneuploidy in cancer cells
Source: Mol Cancer. 2010 Aug 24;9:223. doi: 10.1186/1476-4598-9-223 (PMC2933622; doi:10.1186/1476-4598-9-223)
Supplement: Additional file 2 — Figure S2. Effects of Bcl-3 depletion on apoptosis. Cells with Bcl-3 knockdown are more susceptible to apoptosis induced by ultraviolet light, as assessed by nuclear morphology, viability assays and TUNEL. [file 1476-4598-9-223-S2.PDF]

Additional file 2. Figure S2.

A

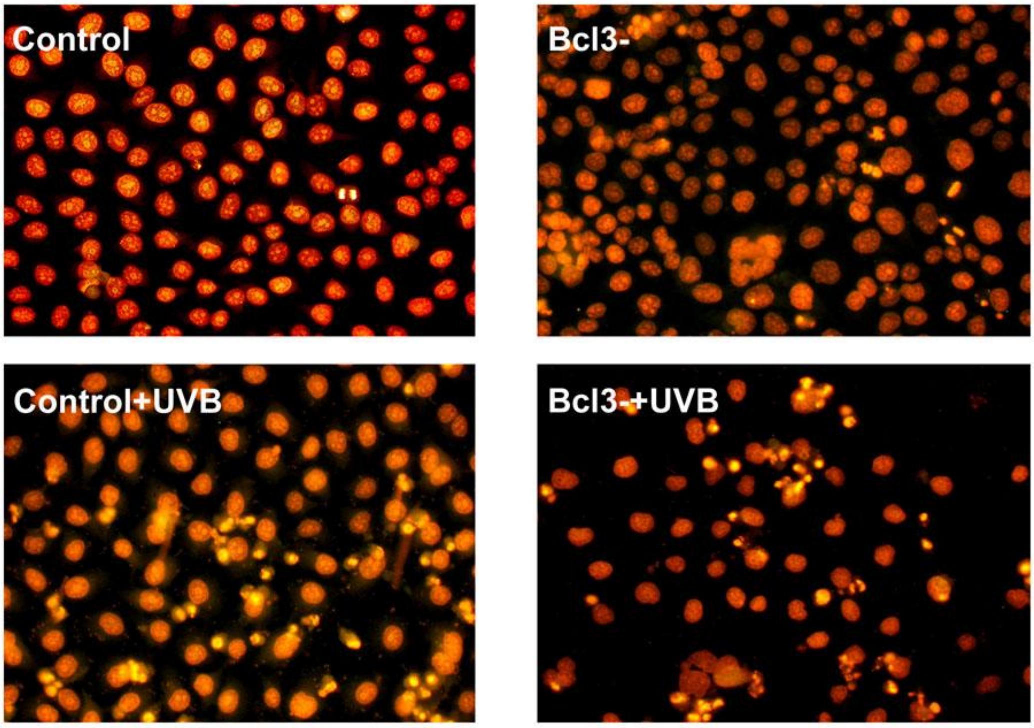

B

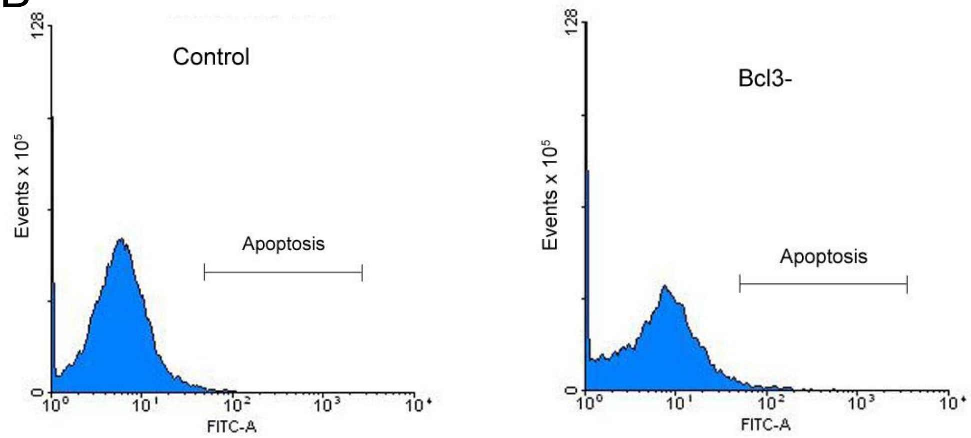

C

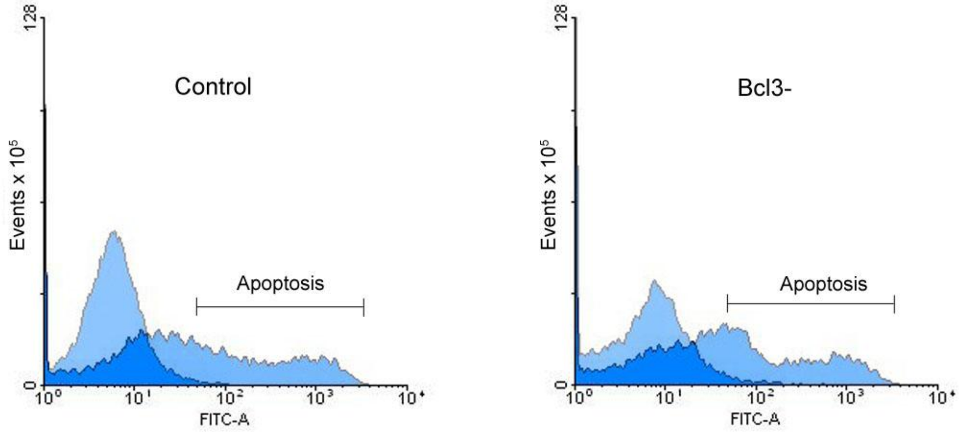

Effects of Bcl-3 depletion on apoptosis. A) HeLa cells stably-transfected with a Bcl-3 shRNA (Bcl3-) or a control shRNA (Control) were exposed to ultraviolet B light (UVB) (280 nm) for 45 seconds. Twenty four hours later, cells were fixed with ethanol (96% for 5 minutes at -20 °C), incubated with RNAse A for 30 minutes, stained with ethidium bromide and visualized with fluorescence microscopy. UVB-exposed cells showed nuclear condensation and fragmentation, typical of apoptosis. A larger number of apoptotic cells were found in Bcl3- cells, even when a large number of apoptotic cells are lost with the staining procedure. In contrast, no difference was found between control and Bcl-3 cells. B) HeLa cells stably-transfected with a Bcl-3 shRNA (Bcl3-) or a control shRNA (Control) were subjected to TUNEL assays. The assay was performed following the manufacturer´s instructions (Apo-Direct; BD Pharmingen, CA; USA). Briefly, cells were fixed with 1 % (w/v) paraformaldehyde in PBS (pH 7.4), and apoptotic cells identified by labeling DNA strand breaks using terminal deoxynucleotidyl transferase (TdT) to transfer FITC-dUTP to these breaks. Cells were then analyzed using a FACSCalibur Flow Cytometer (Becton Dickinson, CA, USA). No difference was found between cell lines. C) TUNEL assays performed on HeLa cells stably-transfected with a Bcl-3 shRNA (Bcl3-) or a control shRNA (Control) exposed to ultraviolet B light (UVB) (280 nm) for 45 seconds. The assay was performed 24 hours after the exposure, as described previously.
