# Supplementary material for: Depletion of the oncoprotein Bcl-3 induces centrosome amplification and aneuploidy in cancer cells
Source: Mol Cancer. 2010 Aug 24;9:223. doi: 10.1186/1476-4598-9-223 (PMC2933622; doi:10.1186/1476-4598-9-223)
Supplement: Additional file 3 — Figure S3. Analysis of Bcl-3 mRNA expression of breast cancer cell lines by RT-PCR. Bcl-3 steady-state levels in breast cancer cell lines with different p53 status (wild type or mutated) and expression. [file 1476-4598-9-223-S3.PDF]

Additional file 3. Figure S3.

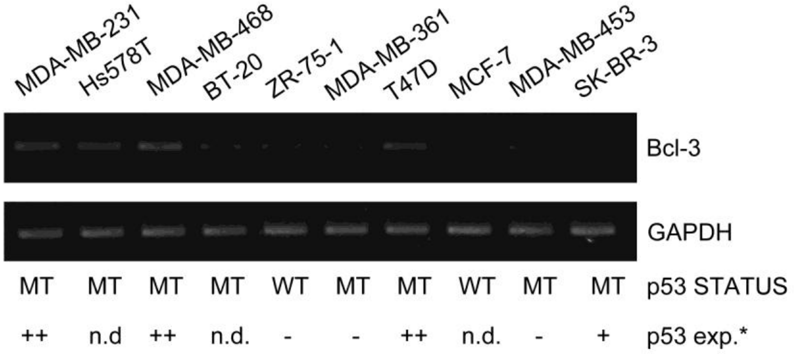

Analysis of Bcl-3 mRNA expression of breast cancer cell lines by end-point RT-PCR. Log cultures of the lines showed were analyzed for Bcl-3 expression, using GAPDH as a loading control. p53 status: WT: Wild type, MT: mutated. p53 exp.: p53 expression. N.d. Not determined. p53 status and expression data obtained from Wasielewski [1]

1. Wasielewski M, Elstrodt F, Klijn JG, Berns EM, Schutte M: **Thirteen new p53 gene mutants identified among 41 human breast cancer cell lines**. *Breast Cancer Res Treat* 2006, **99**(1):97-101.
